# Effects of a postnatal high-salt diet on cardiac function in offspring from hypoxic pregnancies

**DOI:** 10.1098/rstb.2024.0184

**Published:** 2025-08-21

**Authors:** Hayat Baba, Min Zi, Nick Ashton, Gina L. J. Galli

**Affiliations:** ^1^ Division of Cardiovascular Sciences, The Faculty of Biology, Medicine and Health, The University of Manchester, Manchester, UK

**Keywords:** heart, fetal hypoxia, echocardiography, hypertension, high-salt diet, ventricle

## Abstract

Prenatal hypoxia is a common pregnancy complication that can permanently alter the structure and function of the fetal heart and predispose individuals to cardiovascular disease. However, the interactive effects of prenatal hypoxia and postnatal lifestyle choices are poorly studied. This study investigated the combined effects of prenatal hypoxia and a postnatal high-salt diet on cardiac function in adulthood. Pregnant Wistar rats were exposed to either 21% oxygen (normoxic group, *n* = 8) or 10.5% oxygen (hypoxic group, *n* = 8) from days 15 to 20 of gestation. Male offspring were divided into two groups at five weeks of age; normal salt (0.3 % NaCl) and high salt (8% NaCl) chow. Blood pressure and echocardiography measurements were performed at 18 weeks to assess cardiovascular function. Rats from hypoxic pregnancies had signs of pulmonary hypertension and right ventricular thickening, whereas rats fed a postnatal high-salt diet had systemic hypertension, left ventricular wall thickening and impaired relaxation. When prenatal hypoxia was combined with a postnatal high-salt diet, the rats exhibited a combination of the two phenotypes, but there were no significant interactions. Results suggest a poor postnatal diet can put additional strain on the cardiovascular system of offspring from hypoxic pregnancies, which can have significant implications for disease management.

This article is part of the discussion meeting issue ‘Pregnancy at high altitude: the challenge of hypoxia’.

## 1. Introduction

Insufficient oxygen during fetal development (prenatal hypoxia) is a common condition that occurs in a wide range of complicated pregnancies including pre-eclampsia [1,2], reduced fetoplacental perfusion [3] and living at high altitude [4]. In humans, chronic fetal hypoxia triggers the brain-sparing effect which causes asymmetric growth restriction and increased fetal cardiac afterload. If the problem persists, the fetal cardiovascular system remodels, leading to thickening of the cardiac walls and major blood vessels, as well as impaired cardiac output and diastolic filling (reviewed in [5]). These abnormalities persist after birth, and by early childhood, growth retarded offspring from hypoxic pregnancies have increased risk of hypertension [6], heart failure and arrhythmias [7]. However, very little is known about the mechanisms driving cardiovascular dysfunction in these individuals or the interactive effects of postnatal lifestyle choices.

Animal models have shown that adult offspring from hypoxic pregnancies develop pulmonary hypertension with pulmonary arterial wall thickening, decreased pulmonary artery acceleration time and increased right ventricular (RV) diameter in diastole [8–13]. Remodelling of the RV often precipitates left ventricular (LV) failure through increased pulmonary pressure [14]. Indeed, some animal models have also shown offspring from hypoxic pregnancies have LV wall thickening and LV diastolic dysfunction [10,12,15–17]. Systolic function is often unaffected by fetal hypoxia [12], owing to a compensatory increase in sympathetic activity and contractility [16,18]. However, a recent study showed adult offspring from hypoxic pregnancies had systolic dysfunction with reduced contractility [10]. Taken together, animal studies suggest prenatal hypoxia is associated with fetal cardiovascular abnormalities that persist into adulthood, leading to compensated followed by decompensated heart failure.

It is generally accepted that poor postnatal lifestyle choices can exacerbate the long-term effects of intrauterine stress on the cardiovascular system (the so-called ‘second hit’ hypothesis) [19]. In particular, postnatal diets that increase systemic blood pressure (SBP) can place an additional burden on the heart. A classic example is excessive salt intake. Current global salt consumption is more than double the recommended daily limit of 5 g per day [20]. This is a major problem for individuals who are considered salt sensitive and develop hypertension in response to increased salt intake (30−50% of the hypertensive and 25% of the normotensive population) [21,22]. Although the exact mechanism is debatable [23], salt-induced hypertension is associated with water retention, increased systemic peripheral resistance, endothelial dysfunction, changes in the structure and function of large elastic arteries and altered autonomic tone [24]. Some rodent strains are also salt sensitive, including the inbred Dahl rat strain that is genetically susceptible to hypertension in response to a high-salt diet. Other salt-resistant rodents will also develop modest hypertension in response to a diet containing 2–8% w/w salt, including Wistar rats [25,26]. In this strain, a high-salt diet also causes cardiac hypertrophy and interstitial fibrosis [25,27,28]. This could reflect a compensatory mechanism to offset increased systemic vascular resistance, but some of these studies showed high salt could trigger ventricular hypertrophy in the absence of hypertension [29].

Importantly, two studies using CD1 mice have shown a high-salt diet can exacerbate the long-term effects of prenatal hypoxia on the offspring cardiovascular system. At 12 months of age, the combined effects of prenatal hypoxia (12% oxygen from gestational day 14.5 until birth) and a chronic postnatal high-salt diet (5% NaCl in the food) caused significant stiffening of CD1 mouse mesenteric vasculature, loss of aortic elastin integrity and increased aortic collagen, consistent with increased vascular stiffness [30]. In the same model, male offspring from hypoxic pregnancies were more susceptible to salt-induced cardiac and renal fibrosis, which was associated with elevated renal renin and At1aR messenger RNA [31]. Taken together, these studies suggest a postnatal high-salt diet promotes cardiac and vascular fibrosis in offspring from hypoxic pregnancies, which is commonly associated with diastolic dysfunction and the development of heart failure [32].

Given that heart function is already compromised in adult offspring from hypoxic pregnancies [10], we hypothesized that a postnatal high-salt diet may exacerbate ventricular diastolic and systolic dysfunction, leading to cardiac failure. We also expected that a high-salt diet would worsen the ventricular hypertrophy that is commonly observed in male offspring exposed to prenatal hypoxia. Therefore, echocardiography was used to investigate the effects of a high-salt postnatal diet on LV and RV structure and function in adult male rat offspring from hypoxic pregnancies.

## 2. Methods

### (a) Animal model

All procedures comply with the UK Animals (Scientific Procedures) Act 1986 and EU Directive 2010/63 (project license number PD7C22AA9). The ARRIVE guidelines were followed for reporting the use of animals in scientific experiments. Local ethical approval was granted by The University of Manchester Animal Welfare Ethical and Review Board.

A total of 16 pregnant Wistar rats were acquired from Charles River and transported to the University of Manchester on gestational day 6. Rats had free access to standard food and tap water, along with exposure to a 12 L : 12 D cycle. On GD 15, pregnant rats were randomly assigned to one of two groups: hypoxia control (HC, 10.5% O_2_ from GD 15 to 20, *n* = 8) or normoxia control (NC, 21% O_2_ for the whole of gestation, *n* = 8). This model of hypoxia is often used to mimic late-gestation pre-eclampsia, which reduces fetal O_2_ supply as well as nutrient intake (owing to a reduction in maternal food intake) [12,33,34]. For hypoxic exposure, animals were placed into an environmental chamber and nitrogen gas was infused to maintain the oxygen concentration level at 10.5%. Maternal body weight was measured on GD 14 and GD 20, while daily records were maintained for food and water intake throughout gestation. On GD 20, pregnant rats were removed from the chambers and given unrestricted access to food and water until littering. To avoid the confounding effects of maternal nutrition, each litter was reduced to eight pups, with male pups preferentially selected. Sex of the pups was confirmed visually by assessing the anogenital distance.

### (b) Dietary intervention

Offspring were weaned at four weeks of age and randomly assigned to two groups: control food (0.3% NaCl) and high-salt food (8% NaCl). This gave rise to four experimental groups: NC (*n* = 16 pups), normoxic high salt (NHS, *n* = 14 pups), HC (*n* = 14 pups) and hypoxic high salt (HHS, *n* = 15 pups). The diets were purchased commercially from Envigo (high-salt diet, 8% NaCl, catalogue number TD.0314; control diet 0.3% NaCl, catalogue number TD.92101). Rats received the diet for 18−20 weeks, during which time food intake, water consumption and body weight were measured regularly. At the end of the study, rats were culled with CO_2_ exposure and cervical dislocation. Tissues were collected and preserved for further investigation.

### (c) Blood pressure measurements

Blood pressure (BP) was measured at three specific time points: weeks 4 (before the introduction of the diet), 10 and 18. SBP and diastolic blood pressure (DBP) measurements were performed on conscious, unrestrained rats using the tail-cuff method (The CODA noninvasive monitoring BP system, Kent Scientific Corporation). Pulse pressure (PP) and mean blood pressure (MBP) were calculated using the following equation: PP = SBP DBP, MBP = DBP+1/3PP.

### (d) Echocardiography

Transthoracic echocardiography was performed in rats at 20 weeks of age using an Acuson Sequoia C256 system (Siemens) and a 14-MHz probe. Rats were lightly anaesthetized with 1% isoflurane, with heart rate of approximately 300−400 beats per minute. The M-mode parasternal short-axis views were taken to determine LV end-diastolic dimension (LVEDd) and end-systolic dimension (LVEDs), posterior wall thickness in diastole (LVPWd) and systole and interventricular septum thickness in diastole (dIVS) and systole. LV fractional shortening (FS) was calculated using the formula FS = [(LVEDd - LVEDs)/(LVEDd)] × 100. Relative wall thickness (RWT) was calculated using the equation RWT = (dIVS + LVPWd)/LVEDd. The M-mode parasternal long-axis views were taken to determine the RV end-diastolic dimension and end-systolic dimension. Pulse wave Doppler in the parasternal short- and long-axis views was used to measure the peak pulmonary velocity and pressure gradient, and peak aortic velocity and pressure gradient, respectively. Pulse wave Doppler in the apex four chamber view was used to measure transmitral flow velocity to determine LV E wave velocity, A wave velocity, isovolumic contraction time (IVCT), isovolumic relaxation time (IVRT), ejection time (ET) and E wave deceleration time (DT). E/A ratio = E/A (the ratio of the peak velocity of the early (E) wave to the peak velocity of the atrial (A) wave during ventricular filling). The index of myocardial performance (IMP) = (IVCT + IVRT)/ET. The analysis of the RV compact myocardium (RV CM) was performed by measuring the mid-RV diameter across the middle of the RV chamber using an apical four chamber view on echocardiographic images. RV outflow tract analysis was performed using the short-axis subcostal view. The pulsed-wave Doppler sample volume was placed just below the level of the pulmonary valve, at the midpoint of the RV outflow tract. Doppler velocity measurements were recorded from the RV outflow tract to determine two key parameters: AT and ET. The AT/ET ratio was then calculated as an estimate of pulmonary vascular resistance.

Data were averages of measurements over three cardiac cycles, and the technician was blinded to animal codes.

### (e) Statistics

All statistical analysis was carried out on Graphpad and SPSS (IBM). Datasets were tested for normality with a quantile-quantile plot, and all data were found to be normally distributed. The statistical test for each experiment can be found in the associated figure legend. In both graphs and text, data are represented as mean ± s.e.m, and a *p-*value < 0.05 was considered statistically significant.

## 3. Results

### (a) Maternal and neonatal biometry

**Figure 1.**
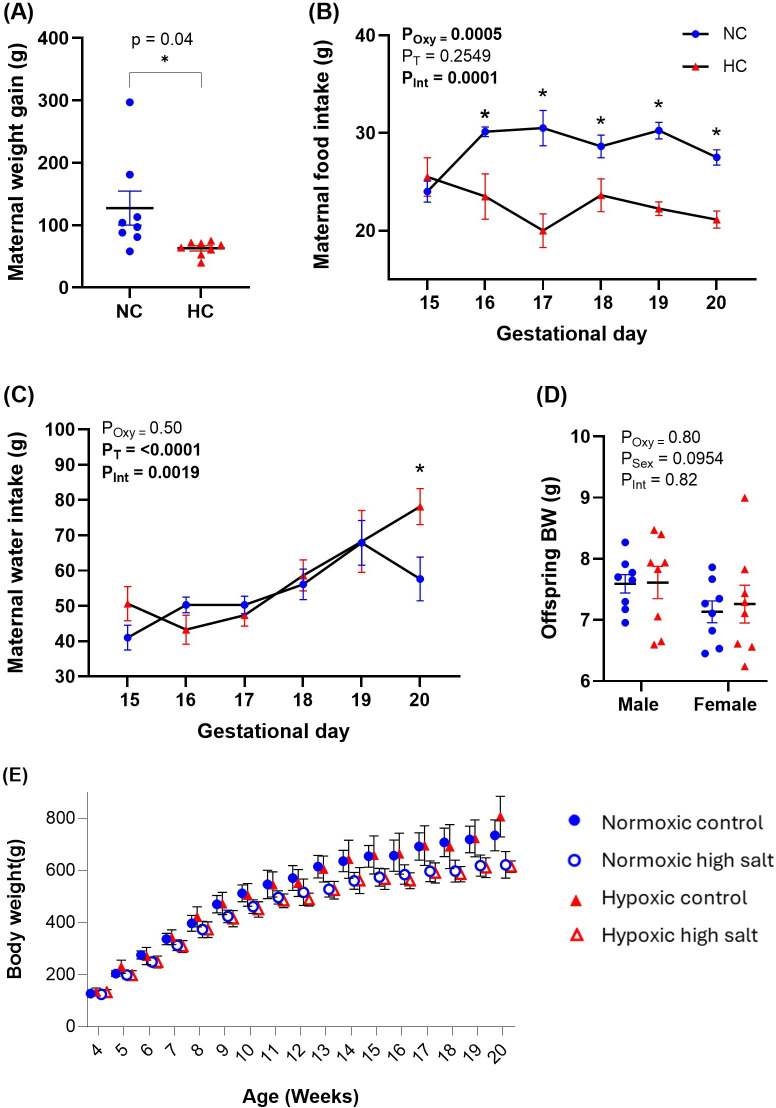
Biometry data for dams and offspring. Maternal weight gain was measured between GD 15 and 20 (A). Food and water were monitored every day in normoxic control (NC, *n* = 8) and hypoxic control (HC, *n* = 8) treated dams (B and C). Offspring body weight (BW) was averaged for each litter on day 1 of littering (D), and weekly between 3-20 weeks (E). Error bars show mean  ±  s.e.m. Significance was assessed using an unpaired *t*‐test (A), a two-way repeated ANOVA (B and C), or a two-way ANOVA (D). Main *p*-values are given in inset (*P*_Oxy_ = effect of oxygen treatment, *P*_T_ = effect of time, *P*_Int_ = interaction between factors, *P*_Sex_, effect of offspring sex). An asterisk, *, signifies a significant difference between normoxic controls and hypoxic controls, *p* < 0.05.

Maternal body weight gain between GD 15 and 20 was significantly lower in the hypoxic-treated dams (figure 1A), and this was associated with a significant reduction in food intake at all time points after GD 15 (figure 1B). Maternal water intake progressively increased across gestation, and there were no differences in experimental groups until GD 20, where hypoxic dams drank more water than controls (figure 1C). Offspring body weight on postnatal day 1 was similar between all experimental groups and sexes (figure 1D).

### (b) Blood pressure measurements

**Figure 2.**
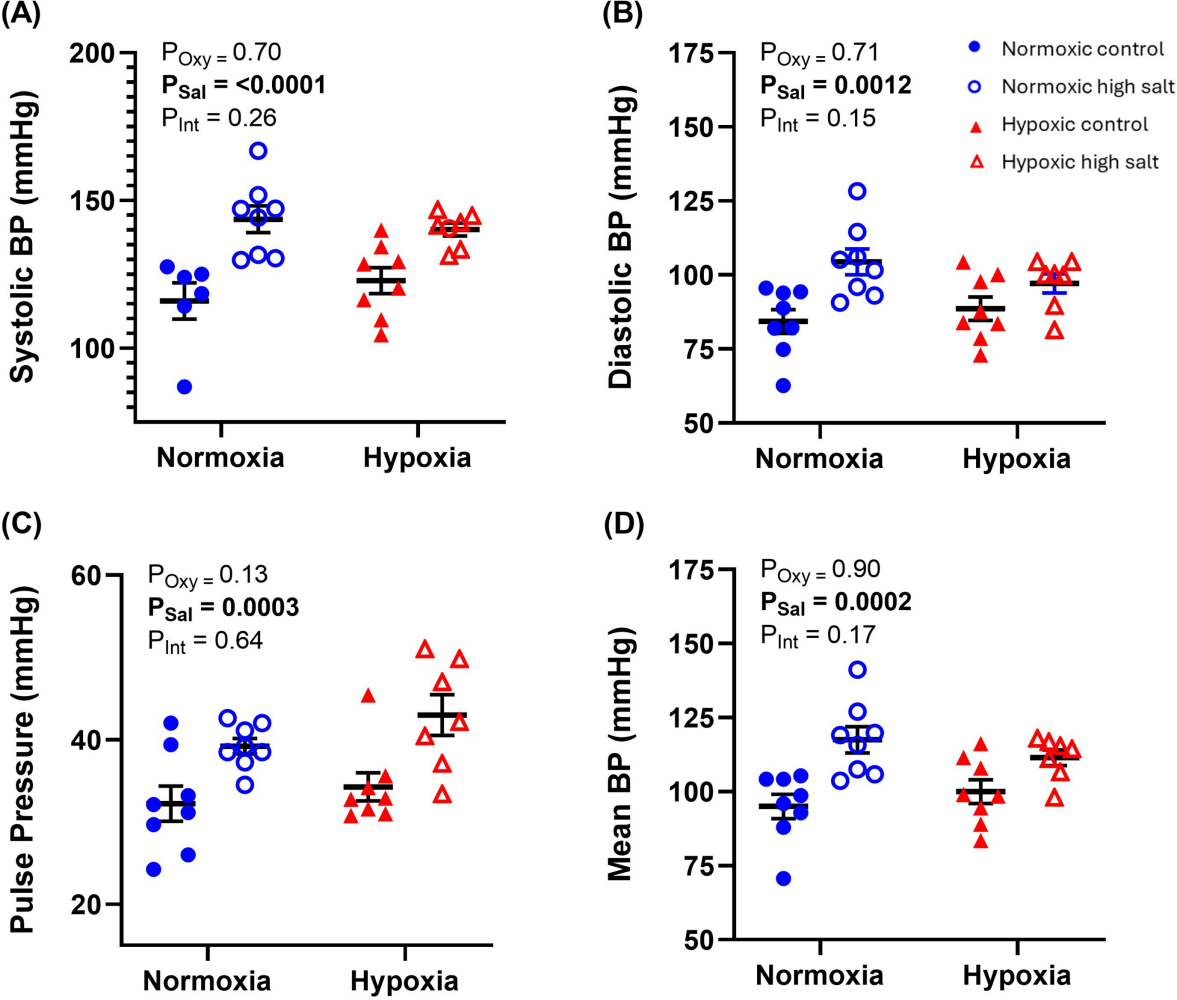
Offspring blood pressure (BP) parameters at 18 weeks. BP at week 18 of offspring from normoxic (blue circle symbols) and hypoxic (red triangle symbols) pregnancies with a postnatal control normal salt (closed symbols) or high-salt (open symbols) diet. (A) SBP, (B) DBP, (C) PP, and (D) MBP. Error bars show mean  ±  s.e.m. Each experimental group had *n* = 8 offspring from eight pregnancies. Significance was assessed using a two-way ANOVA (factors: oxygen and salt treatment). No significant interactions were observed between prenatal hypoxia and a high-salt diet, so a *post hoc* test was not performed. The *p*-values for main effects are given in inset (*P*_Oxy_ = effect of prenatal oxygen treatment, *P*_Sal_ = effect of postnatal salt, *P*_Int_ = interaction between factors). Asterisks, * and ** signify *p* < 0.05 and *p* < 0.01, respectively.

There was no effect of a postnatal high-salt diet or hypoxia on BP in 4- or 10-week-old rats (electronic supplementary material, table S1). However, at 18 weeks, a high-salt postnatal diet increased systolic pressure, diastolic pressure, PP and MBP (figure 2). This effect was apparent in both experimental groups, but the interaction between postnatal salt and prenatal oxygen treatment was not significant (figure 2 insets).

### (c) Echocardiography measurements

**Figure 3.**
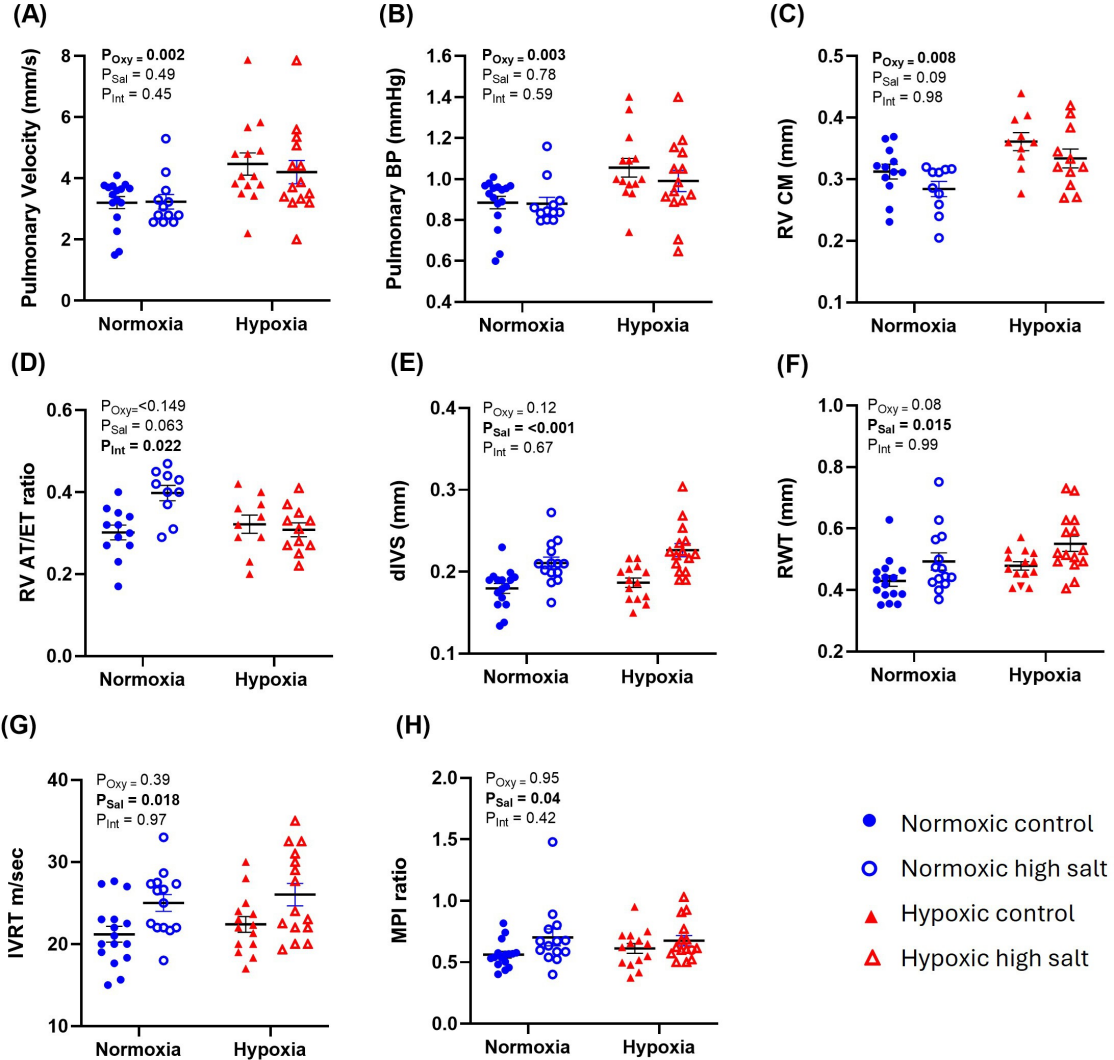
Offspring echocardiography data. Echocardiography was performed at week 18 in offspring from normoxic (blue round symbols) and hypoxic (red triangle symbols) pregnancies with a postnatal low-salt (closed symbols) or high-salt (open symbols) diet. (A) Pulmonary velocity, (B) pulmonary blood pressure (BP), (C) right ventricular (RV) compact myocardium (RV CM), (D) the pulmonary arterial acceleration time/ejection time (AT/ET ratio), (E) dimensions of the interventricular septum at end diastole (dIVS), (F) LV relative wall thickness (RWT), (G) LV isovolumic relaxation time (IVRT), (H) myocardial performance index (MPI). Error bars show mean  ±  s.e.m. Statistical significance: linear mixed modelling was performed to account for the nested (clustered) design of the experiment, e.g. multiple observations from the same animal. No significant interactions were observed between prenatal hypoxia and a high-salt diet, so a *post hoc* test was not performed. The *p*-values for main effects are given in inset (*P*_Oxy_ = effect of prenatal oxygen treatment, *P*_Sal_ = effect of postnatal salt treatment, *P*_Int_ = interaction between factors). Asterisks, *, ** and *** signify *p* < 0.05, *p* < 0.01 and *p* < 0.0001, respectively. For each experimental group, *n* = 8 pregnancies, with 1 or 2 pups from each litter (normoxic control, *n* = 16 pups; hypoxic control, *n* = 14 pups; normoxic high salt, *n* = 14 pups; and hypoxic high salt *n* = 15 pups.

Adult offspring exposed to prenatal hypoxia had signs of pulmonary hypertension and RV failure, including increased pulmonary pressure and velocity (figure 3A,B) and RV wall thickening (figure 3C). This effect was evident in both groups of rats from control and high-salt diets, and the effect was not exacerbated by a high-salt diet (i.e. there was no interaction between treatments (see insets in figure 3A–C)). A high-salt diet led to LV hypertrophy (increased dIVS and RWT; figure 3E,F), as well as prolonged IVRT and an increased myocardial performance index (MPI; figure 3G,H). These effects were evident in rats from normoxic and hypoxic cohorts, and they were not exacerbated by prenatal hypoxia (see insets in figure 3D–G). All the other echocardiography parameters were not affected by either treatment and are displayed in table 1.

**Table 1.** Offspring echocardiography parameters at 18 weeks. (Abbreviations: NC, normoxic control; NHS, normoxic high salt; HC, hypoxic control; HHS, hypoxic high salt; BP, blood pressure. In all experimental groups, offspring were from eight pregnancies: NC (*n* = 16 pups), HC (*n* = 14 pups), NHS (*n* = 14 pups) and HHS (*n* = 15 pups). Data are mean ± s.e.m. To test for statistical significance, a linear mixed model was performed to account for the nested (clustered) design of the experiment. *P*-values are given in the final columns. Bold text signifies a significant main effect or interaction. Abbreviations: Heart rate, HR; Pulmonary, Pul; Interventricular septal thickness, IVST; Diastolic prosterior wall thickness, dPW.

	NC	NHS	HC	HHS	***P*-values**		
					*P*_Oxy_	*P*_Sal_	*P*_Int_
HR (BPM)	315.2 ± 23.2	289.7 ± 23.4	303.9 ± 26.0	303.0 ± 24.5	0.19	0.22	0.14
Pul velocity (m ms^−1^)	3.21 ± 0.20	3.23 ± 0.23	**4.47 ± 0.36**	**4.20 ± 0.38**	**0.002**	0.49	0.45
Pul BP (mmHg)	0.88 ± 0.03	0.88 ± 0.03	**1.06 ± 0.03**	**0.99 ± 0.05**	**0.003**	0.78	0.59
RV CM (mm)	0.31 ± 0.01	0.28 ± 0.01	**0.36 ± 0.01**	**0.33 ± 0.02**	**0.008**	0.09	0.98
RV FS%	85.55 ± 5.75	51.57 ± 11.73	44.99 ± 22.82	36.51 ± 5.47	**<0.001**	0.001	0.733
S/D ratio	2.92 ± 0.75	2.51 ± 0.81	2.30 ± 1.28	2.22 ± 1.16	**0.153**	0.428	0.605
DT (ms)	32.5 ± 2.3	29.6 ± 2.6	26.9 ± 1.6	27.9 ± 1.0	0.12	0.57	0.36
E/A ratio	1.24 ± 0.08	1.17 ± 0.08	1.28 ± 0.09	1.28 ± 0.12	0.49	0.77	0.63
IVRT (m s^−1^)	21.2 ± 1.0	**25.0 ± 1.0**	22.4 ± 1.0	**26.0 ± 1.4**	0.39	**0.018**	0.97
IVST (m s^−1^)	14.2 ± 1.1	17.1 ± 1.6	14.4 ± 1.5	15.3 ± 1.0	0.76	0.17	0.63
LV FS (%)	51.8 ± 1.4	47.7 ± 1.4	48.7 ± 1.1	51.2 ± 1.8	0.90	0.58	**0.03**
MPI ratio	0.58 ± 0.03	**0.70 ± 0.07**	0.64 ± 0.04	**0.68 ± 0.04**	0.95	**0.039**	0.42
ET (m s^−1^)	63.6 ± 1.6	63.5 ± 3.4	61.2 ± 1.9	62.1 ± 1.9	0.87	0.47	0.60
dIVS (mm)	0.18 ± 0.01	**0.21 ± 0.01**	0.19 ± 0.01	**0.23 ± 0.01**	0.12	**<0.001**	0.67
dPW (mm)	0.18 ± 0.01	0.20 ± 0.01	0.20 ± 0.01	0.21 ± 0.01	0.3	0.092	0.42
RWT (mm)	0.42 ± 0.01	**0.49 ± 0.03**	0.48 ± 0.02	**0.55 ± 0.02**	0.08	**0.015**	0.99
AT/ET ratio	0.351 ± 0.016	0.312 ± 0.016	0.317 ± 0.016	0.356 ± 0.016	0.149	0.063	**0.022**

## 4. Discussion

Our study is, to our knowledge, the first to assess the interactive effects of prenatal hypoxia and a postnatal high-salt diet on cardiac function. Similar to previous studies, we show that prenatal hypoxia leads to pulmonary hypertension in adult offspring, characterized by elevated pulmonary pressure, increased pulmonary blood flow velocity and thickening of the RV wall. When offspring from hypoxic pregnancies were fed a high-salt diet, they also developed systemic hypertension, LV hypertrophy and diastolic dysfunction. There was no statistical interaction between these effects, meaning that a high-salt diet did not exacerbate pulmonary hypertension in offspring from hypoxic pregnancies, and prenatal hypoxia did not exacerbate systemic hypertension and LV failure in offspring fed a high-salt diet. Taken together, our data suggest a high-salt diet can cause bidirectional ventricular failure in offspring from hypoxic pregnancies in early adulthood.

### (a) Effects of prenatal hypoxia alone

While prenatal hypoxia reduced maternal food intake and weight gain during pregnancy, it did not cause intrauterine growth restriction (IUGR) in the fetuses. This is in contrast to previous studies using similar models (10.5–12% oxygen, GD 15−20), which found rodent offspring have smaller body weights at birth and larger heart/body weight ratios than controls [12,33,35]. While IUGR is common in human pregnancies complicated with hypoxia, it is not universal. For example, IUGR occurs in 15–30% of pre-eclamptic pregnancies, and it is less likely in late-gestation pre-eclampsia, which our rat model most closely resembles [2]. In our study, it is possible that hypoxia caused a compensatory increase in placental growth, which is known to maintain fetal growth in rodent models with milder levels of hypoxia (>13% oxygen [36–39]).

In agreement with other studies on animals [9,10,12,40–42] and humans [43], we found that offspring from hypoxic pregnancies had signs of pulmonary hypertension, including increased pulmonary velocity and pulmonary pressure gradient. Pulmonary hypertension affects 1% of the global population and up to 10% of individuals aged over 65 [44]. However, the AT/ET ratio, which is an accepted estimate of pulmonary resistance, was not affected by fetal hypoxia. This may indicate that the heart is in an early, compensated phase of pulmonary hypertension, where the right ventricle initially enlarges as a compensatory mechanism to increase contractility and overcome the increased afterload caused by elevated pulmonary pressure [14]. Indeed, we have shown here and previously [10] that offspring from hypoxic pregnancies have RV wall thickening. Nevertheless, to confirm the diagnosis of compensated pulmonary hypertension, it is important to perform the longitudinal systolic assessment of the RV with tricuspid annular plane systolic excursion and other variables such as fractional area change and peak systolic velocity of the tricuspid annulus (S’ wave). This limitations of our study could be addressed in future work.

Although initially adaptive, RV remodelling can precipitate LV failure via increased pulmonary pressure, augmented LV workload, impaired filling and systemic compensatory mechanisms [14]. Eventually, LV end-diastolic volume and mass decrease, and stroke volume and ejection fraction decline, leading to bidirectional heart failure. While LV dysfunction has previously been observed alongside pulmonary hypertension in offspring from hypoxic pregnancies [10,12,45], we did not observe any changes in LV structure or function in the present study. This suggests rats were at an early stage of pulmonary hypertension where the right ventricle was compensated, and the left ventricle was unaffected.

The lack of LV dysfunction in hypoxic offspring could be partly owing to our experimental design. For example, some studies use milder levels of hypoxia that start earlier in gestation (GD 6−20), which may affect LV development differently [10]. In that model, four-month-old rats exhibited LV and RV hypertrophy, as well as systolic and diastolic dysfunction in both ventricles [10]. In another study that used a similar model to ours (12% oxygen from GD 15−20), offspring had LV hypertrophy, diastolic dysfunction and pulmonary hypertension; but it was only evident at 12 months of age and not four months [12]. These findings emphasize a progressive deterioration of the hypoxia phenotype with age, suggesting that rats in the present study may have gone on to develop LV dysfunction later in life.

Similar to a previous study that measured BP in rats with implantable telemetry probes [46], prenatal hypoxia had no effect on SBP or DBP at 4−18 weeks of age. This is despite previous reports of vascular remodelling and heightened vasoreactivity within this age range [47]. Nevertheless, several studies using tail-cuff measurements and implantable telemetry probes have shown systemic hypertension emerges in middle-aged (6–12 months old) rodents [30,48–51] and sheep [52] from hypoxic pregnancies, which is often accompanied by endothelial dysfunction and structural abnormalities in blood vessels [30,47,53]. There is also a well-established correlation between low birth rate and the development of hypertension in adulthood in humans [54]. Therefore, prenatal hypoxia appears to have latent effects on the systemic vasculature, leading to an increased risk of hypertension in later life. This supports the idea that there is some physiological reserve in early life, but aging may act as a ‘second hit’, revealing underlying circulatory dysfunction. Nevertheless, it should be noted that our study measured BP with the tail-cuff method, which can be less accurate than *in vivo* techniques [55]. Future studies should use implantable telemeters to assess the effects of prenatal hypoxia on BP and ideally extend the measurements to 12 months.

### (b) Effects of postnatal high-salt diet alone

A postnatal high-salt diet alone triggered systemic hypertension, LV hypertrophy and an increase in IVRT in Wistar rats aged 18 weeks. While this strain is considered to be salt-resistant, previous studies have also demonstrated modest hypertension with NaCl concentrations between 2 and 8% ww [26,27,56]. Long-term hypertension is associated with an increased risk of conditions affecting the cardiovascular, cerebrovascular and renal systems, significantly contributing to premature mortality [57–59]. While the mechanisms remain controversial, salt-induced hypertension is associated with water retention, remodelling of small resistant arteries, autonomic nerve activity, changes in the structure and function of large elastic arteries and endothelial dysfunction [60].

In agreement with other studies on Wistar rats [25,27,28], we found that a high-salt diet caused LV hypertrophy at 18 weeks. This could reflect a compensatory mechanism to offset increased systemic vascular resistance, similar to ventricular remodelling in human hypertensive disorders [61]. However, some studies in Wistar rats suggest salt may also cause ventricular hypertrophy in the absence of hypertension [29]. Several non-haemodynamic factors have been proposed to be involved, including angiotensin signalling, oxidative stress and proinflammatory cytokines [62]. Although initially compensatory, hypertrophic remodelling is often followed by a decompensation phase, with diastolic dysfunction, chamber dilation and ultimately heart failure [61]. Indeed, we also found that rats on a high-salt diet had a reduced IVRT, which indicates impaired ventricular relaxation. Given that the E/A ratio was normal, this could simply be explained by an increased afterload (higher BP). However, it could also represent a grade II pattern of diastolic dysfunction whereby a rise in left atrial pressure artificially increases the E wave velocity, masking the underlying impairment in LV relaxation and filling (known as pseudonormalization) [63]. In support of this contention, Dahl salt-sensitive rats fed an 8% NaCl diet had a reduced E/A ratio after 14 weeks, but it normalized after 18 weeks owing to pseudonormalization [64]. If diastolic dysfunction is occurring with high salt, it could be driven by LV interstitial fibrosis as well as perivascular fibrosis in intramyocardial coronary arteries, which was previously observed in spontaneously hypertensive rats and normotensive Wistar-Kyoto rats [65].

### (c) Interactive effects of prenatal hypoxia and high-salt diet

When prenatal hypoxia and a postnatal high-salt diet were combined, offspring had a combination of the individual phenotypes described above, with systemic and pulmonary hypertension, LV and RV thickening, reduced RV contractility, diastolic dysfunction and a reduced MPI. However, we did not find any statistically significant interactive effects between prenatal hypoxia and a postnatal high-salt diet. In other words, a high-salt diet did not exacerbate pulmonary hypertension in offspring from hypoxic pregnancies, and prenatal hypoxia did not exacerbate systemic hypertension and LV failure in offspring fed a high-salt diet. This suggests the two insults are causing distinct cardiovascular effects via different cellular and molecular pathways.

Very few studies have examined the interactive effects of prenatal hypoxia and a postnatal high-salt diet on the cardiovascular system, and those that have primarily focussed on vascular structure and function, as well as structural changes in the heart. In one study, 12-month-old mice exposed to prenatal hypoxia (12% oxygen, GD 15−20) had mild endothelial dysfunction, but when it was combined with a high-salt postnatal diet (5% NaCl), offspring had mesenteric artery stiffening and changes in the extracellular matrix composition of the aorta, including a marked loss of elastin fibre integrity [30]. In another study using the same model, prenatal hypoxia resulted in cardiac and renal impairments, including glomerulosclerosis and hypertrophy [31], and this was exacerbated by a high-salt diet. It is possible that we would have observed more interactions between prenatal hypoxia and a high-salt diet if the offspring were reared to 12 months.

## 5. Conclusion and future directions

In conclusion, the effects of prenatal hypoxia and a postnatal high-salt diet on cardiac function were distinct and presumably driven by separate mechanisms. Offspring from hypoxic pregnancies had signs of pulmonary hypertension and RV remodelling, while a postnatal high-salt diet resulted in systemic hypertension and LV remodelling with impaired diastolic relaxation. Therefore, the combination of these stressors leads to biventricular dysfunction, which may predispose individuals to cardiovascular disease later in life. This condition is likely to worsen with age because several studies have shown rodent offspring from hypoxic pregnancies eventually develop LV dysfunction in response to RV hypertrophy. Future work should be focused on understanding the interactive effects of prenatal hypoxia and postnatal diet in the later stages of life, as well as identifying the underlying pathophysiological mechanisms.

## Data Availability

The data supporting the findings reported in this article are openly available as electronic supplementary material from the Figshare repository at [66]. Supplementary material is available online [67].

## References

[1] 2000 Report of the national high blood pressure education program working group on high blood pressure in pregnancy. American Journal of Obstetrics and Gynecology, pp. S1–S22. (10.1067/mob.2000.107928) 10920346

[2] Sibai BM. 2003 Diagnosis and management of gestational hypertension and preeclampsia. Obstet. Gynecol. **102**, 181–192. (10.1016/s0029-7844(03)00475-7) 12850627

[3] Krebs C, Macara LM, Leiser R, Bowman AW, Greer IA, Kingdom JC. 1996 Intrauterine growth restriction with absent end-diastolic flow velocity in the umbilical artery is associated with maldevelopment of the placental terminal villous tree. Am. J. Obstet. Gynecol. **175**, 1534–1542. (10.1016/s0002-9378(96)70103-5) 8987938

[4] Giussani DA, Spencer JA, Moore PJ, Bennet L, Hanson MA. 1993 Afferent and efferent components of the cardiovascular reflex responses to acute hypoxia in term fetal sheep. J. Physiol. **461**, 431–449. (10.1113/jphysiol.1993.sp019521) 8350271 PMC1175265

[5] Giussani DA. 2016 The fetal brain sparing response to hypoxia: physiological mechanisms. J. Physiol. **594**, 1215–1230. (10.1113/jp271099) 26496004 PMC4721497

[6] Zanardo V, Visentin S, Trevisanuto D, Bertin M, Cavallin F, Cosmi E. 2013 Fetal aortic wall thickness: a marker of hypertension in IUGR children? Hypertens. Res. **36**, 440–443. (10.1038/hr.2012.219) 23364342

[7] Nahum Sacks K, Friger M, Shoham-Vardi I, Spiegel E, Sergienko R, Landau D, Sheiner E. 2018 Prenatal exposure to preeclampsia as an independent risk factor for long-term cardiovascular morbidity of the offspring. Pregnancy Hypertens. **13**, 181–186. (10.1016/j.preghy.2018.06.013) 30177050

[8] Botting KJ *et al*. 2020 Translatable mitochondria-targeted protection against programmed cardiovascular dysfunction. Sci. Adv. **6**, eabb1929. (10.1126/sciadv.abb1929) 32875110 PMC7438086

[9] Ding H, Luo Y, Hu K, Huang H, Liu P, Xiong M, Zhu L, Yi J, Xu Y. 2020 Hypoxia in utero increases the risk of pulmonary hypertension in rat offspring and is associated with vasopressin type‑2 receptor upregulation. Mol. Med. Rep. **22**, 4173–4182. (10.3892/mmr.2020.11533) 33000260 PMC7533485

[10] Lock MC, Patey OV, Smith KLM, Niu Y, Jaggs B, Trafford AW, Giussani DA, Galli GLJ. 2024 Maladaptive cardiomyocyte calcium handling in adult offspring of hypoxic pregnancy: protection by antenatal maternal melatonin. J. Physiol. **602**, 6683–6703. (10.1113/JP287325) 39572933 PMC11649517

[11] Li H, Ji B, Xu T, Zhao M, Zhang Y, Sun M, Xu Z, Gao Q. 2021 Antenatal hypoxia affects pulmonary artery contractile functions via downregulating L‐type Ca2+ channels subunit alpha1 C in adult male offspring. J. Am. Heart Assoc. **10**, e019922. (10.1161/jaha.120.019922) 33843249 PMC8174167

[12] Rueda-Clausen CF, Morton JS, Davidge ST. 2009 Effects of hypoxia-induced intrauterine growth restriction on cardiopulmonary structure and function during adulthood. Cardiovasc. Res. **81**, 713–722. (10.1093/cvr/cvn341) 19088083

[13] Skeffington KL, Beck C, Itani N, Niu Y, Shaw CJ, Giussani DA. 2020 Hypertension programmed in adult hens by isolated effects of developmental hypoxia in ovo. Hypertension **76**, 533–544. (10.1161/hypertensionaha.120.15045) 32536277 PMC7340221

[14] Rosenkranz S, Howard LS, Gomberg-Maitland M, Hoeper MM. 2020 Systemic consequences of pulmonary hypertension and right-sided heart failure. Circulation **141**, 678–693. (10.1161/circulationaha.116.022362) 32091921

[15] Aljunaidy MM, Morton JS, Kirschenman R, Phillips T, Case CP, Cooke CLM, Davidge ST. 2018 Maternal treatment with a placental-targeted antioxidant (MitoQ) impacts offspring cardiovascular function in a rat model of prenatal hypoxia. Pharmacol. Res. **134**, 332–342. (10.1016/j.phrs.2018.05.006) 29778808

[16] Niu Y *et al*. 2018 Maternal allopurinol prevents cardiac dysfunction in adult male offspring programmed by chronic hypoxia during pregnancy. Hypertension **72**, 971–978. (10.1161/hypertensionaha.118.11363) 30354714 PMC6135482

[17] Spiroski A *et al*. 2021 Mitochondria antioxidant protection against cardiovascular dysfunction programmed by early‐onset gestational hypoxia. FASEB J. **35**, e21446. (10.1096/fj.202002705r) 33788974 PMC7612077

[18] Hauton D, Ousley V. 2009 Prenatal hypoxia induces increased cardiac contractility on a background of decreased capillary density. BMC Cardiovasc. Disord. **9**, 1. (10.1186/1471-2261-9-1) 19126206 PMC2627821

[19] Thornburg KL. 2015 The programming of cardiovascular disease. J. Dev. Orig. Health Dis. **6**, 366–376. (10.1017/s2040174415001300) 26173733 PMC7587080

[20] Tan M. 2024 Mandatory salt targets: a key policy tool for global salt reduction efforts. Lancet Public Health **9**, e5. (10.1016/S2468-2667(24)00227-5) 39486898

[21] Balafa O, Kalaitzidis RG. 2021 Salt sensitivity and hypertension. J. Hum. Hypertens **35**, 184–192. (10.1038/s41371-020-00407-1) 32862203

[22] Elijovich F *et al*. 2016 Salt sensitivity of blood pressure. Hypertension **68**, e7–e46. (10.1161/HYP.0000000000000047) 27443572

[23] Grillo A, Salvi L, Coruzzi P, Salvi P. 2019 Sodium intake and hypertension. Nutrients **11**, 1970. (10.3390/nu11091970) 31438636 PMC6770596

[24] Cowley Jr A. 1991 Salt and hypertension-future directions. Hypertension **17**, I205–10. (10.1161/01.hyp.17.1_suppl.i205) 1987005

[25] Katayama IA, Pereira RC, Dopona EPB, Shimizu MHM, Furukawa LNS, Oliveira IB, Heimann JC. 2014 High-salt intake induces cardiomyocyte hypertrophy in rats in response to local angiotensin II type 1 receptor activation. J. Nutr. **144**, 1571–1578. (10.3945/jn.114.192054) 25122644

[26] Gomes PM, Batista JS, Sá RWM, Antunes VR. 2023 Short exposure to high salt in drinking solution leads to a cardiovascular phenotype of hypertension without changes in the blood volume of rats. Exp. Physiol. **108**, 361–370. (10.1113/ep090912) 36715005 PMC10103861

[27] Ferreira DN, Katayama IA, Oliveira IB, Rosa KT, Furukawa LNS, Coelho MS, Casarini DE, Heimann JC. 2010 Salt-induced cardiac hypertrophy and interstitial fibrosis are due to a blood pressure–independent mechanism in Wistar rats. J. Nutr. **140**, 1742–1751. (10.3945/jn.109.117473) 20724490

[28] Lal A. 2003 Prevention of high salt diet-induced cardiac hypertrophy and fibrosis by spironolactone. Am. J. Hypertens. **16**, 319–323. (10.1016/s0895-7061(02)03268-5) 12670750

[29] Burnier M, Phan O, Wang Q. 2007 High salt intake: a cause of blood pressure-independent left ventricular hypertrophy? Nephrol. Dial. Transplant. **22**, 2426–2429. (10.1093/ndt/gfm321) 17556416

[30] Walton SL, Singh RR, Tan T, Paravicini TM, Moritz KM. 2016 Late gestational hypoxia and a postnatal high salt diet programs endothelial dysfunction and arterial stiffness in adult mouse offspring. J. Physiol. **594**, 1451–1463. (10.1113/jp271067) 26456386 PMC4771779

[31] Walton SL, Bielefeldt-Ohmann H, Singh RR, Li J, Paravicini TM, Little MH, Moritz KM. 2017 Prenatal hypoxia leads to hypertension, renal renin-angiotensin system activation and exacerbates salt-induced pathology in a sex-specific manner. Sci. Rep. **7**, 8241. (10.1038/s41598-017-08365-4) 28811528 PMC5557956

[32] Travers JG, Kamal FA, Robbins J, Yutzey KE, Blaxall BC. 2016 Cardiac fibrosis. Circ. Res. **118**, 1021–1040. (10.1161/circresaha.115.306565) 26987915 PMC4800485

[33] Rueda-Clausen CF, Dolinsky VW, Morton JS, Proctor SD, Dyck JRB, Davidge ST. 2011 Hypoxia-induced intrauterine growth restriction increases the susceptibility of rats to high-fat diet–induced metabolic syndrome. Diabetes **60**, 507–516. (10.2337/db10-1239) 21270262 PMC3028350

[34] Rueda-Clausen CF, Morton JS, Dolinsky VW, Dyck JRB, Davidge ST. 2012 Synergistic effects of prenatal hypoxia and postnatal high-fat diet in the development of cardiovascular pathology in young rats. Am. J. Physiol. Regul. Integr. Comp. Physiol. **303**, R418–R426. (10.1152/ajpregu.00148.2012) 22739349

[35] de Grauw TJ, Myers RE, Scott WJ. 1986 Fetal growth retardation in rats from different levels of hypoxia. Biol. Neonate **49**, 85–89. (10.1159/000242515) 3697431

[36] Smith KLM *et al*. 2022 Chronic developmental hypoxia alters mitochondrial oxidative capacity and reactive oxygen species production in the fetal rat heart in a sex‐dependent manner. J. Pineal Res. **73**, e12821. (10.1111/jpi.12821) 35941749 PMC9540814

[37] Giussani DA *et al*. 2012 Developmental programming of cardiovascular dysfunction by prenatal hypoxia and oxidative stress. PLoS ONE **7**, e31017. (10.1371/journal.pone.0031017) 22348036 PMC3278440

[38] Nuzzo AM *et al*. 2018 Placental adaptation to early-onset hypoxic pregnancy and mitochondria-targeted antioxidant therapy in a rodent model. Am. J. Pathol. **188**, 2704–2716. (10.1016/j.ajpath.2018.07.027) 30248337 PMC6284551

[39] Richter HG *et al*. 2012 Ascorbate prevents placental oxidative stress and enhances birth weight in hypoxic pregnancy in rats. J. Physiol. **590**, 1377–1387. (10.1113/jphysiol.2011.226340) 22289909 PMC3382329

[40] Herrera EA *et al*. 2010 Long-term exposure to high-altitude chronic hypoxia during gestation induces neonatal pulmonary hypertension at sea level. Am. J. Physiol. Regul. Integr. Comp. Physiol. **299**, R1676–R1684. (10.1152/ajpregu.00123.2010) 20881096 PMC3007194

[41] Papamatheakis DG, Chundu M, Blood AB, Wilson SM. 2013 Prenatal programming of pulmonary hypertension induced by chronic hypoxia or ductal ligation in sheep. Pulm. Circ. **3**, 757–780. (10.1086/674767) 25006393 PMC4070839

[42] Caslin A, Heath D, Smith P. 1991 Influence of hypobaric hypoxia in infancy on the subsequent development of vasoconstrictive pulmonary vascular disease in the Wistar albino rat. J. Pathol. **163**, 133–141. (10.1002/path.1711630209) 1901909

[43] Heath-Freudenthal A *et al*. 2022 Vascular disorders of pregnancy increase susceptibility to neonatal pulmonary hypertension in high-altitude populations. Hypertension **79**, 1286–1296. (10.1161/hypertensionaha.122.19078) 35437031 PMC9098686

[44] Hoeper MM, Humbert M, Souza R, Idrees M, Kawut SM, Sliwa-Hahnle K, Jing ZC, Gibbs JSR. 2016 A global view of pulmonary hypertension. Lancet Respir. Med. **4**, 306–322. (10.1016/s2213-2600(15)00543-3) 26975810

[45] Kumar P *et al*. 2020 Intrauterine exposure to chronic hypoxia in the rat leads to progressive diastolic function and increased aortic stiffness from early postnatal developmental stages. Physiol. Rep. **8**, e14327. (10.14814/phy2.14327) 31960611 PMC6971413

[46] Li X *et al*. 2019 Prenatal hypoxia plus postnatal high‐fat diet exacerbated vascular dysfunction via up‐regulated vascular Cav1.2 channels in offspring rats. J. Cell. Mol. Med. **23**, 1183–1196. (10.1111/jcmm.14020) 30556291 PMC6349350

[47] Williams SJ, Hemmings DG, Mitchell JM, McMillen IC, Davidge ST. 2005 Effects of maternal hypoxia or nutrient restriction during pregnancy on endothelial function in adult male rat offspring. J. Physiol. **565**, 125–135. (10.1113/jphysiol.2005.084889) 15774515 PMC1464495

[48] Rook W, Johnson CD, Coney AM, Marshall JM. 2014 Prenatal hypoxia leads to increased muscle sympathetic nerve activity, sympathetic hyperinnervation, premature blunting of neuropeptide Y signaling, and hypertension in adult life. Hypertension **64**, 1321–1327. (10.1161/hypertensionaha.114.04374) 25267800

[49] Peyronnet J, Dalmaz Y, Ehrström M, Mamet J, Roux JC, Pequignot JM, Thorén HP, Lagercrantz H. 2002 Long-lasting adverse effects of prenatal hypoxia on developing autonomic nervous system and cardiovascular parameters in rats. Pflugers Arch. **443**, 858–865. (10.1007/s00424-001-0766-9) 11889586

[50] Svitok P, Molcan L, Stebelova K, Vesela A, Sedlackova N, Ujhazy E, Mach M, Zeman M. 2016 Prenatal hypoxia in rats increased blood pressure and sympathetic drive of the adult offspring. Hypertens. Res. **39**, 501–505. (10.1038/hr.2016.21) 26911229

[51] Bourque SL, Gragasin FS, Quon AL, Mansour Y, Morton JS, Davidge ST. 2013 Prenatal hypoxia causes long-term alterations in vascular endothelin-1 function in aged male, but not female, offspring. Hypertension **62**, 753–758. (10.1161/hypertensionaha.113.01516) 23940196

[52] Brain KL *et al*. 2019 Intervention against hypertension in the next generation programmed by developmental hypoxia. PLoS Biol. **17**, e2006552. (10.1371/journal.pbio.2006552) 30668572 PMC6342530

[53] Dharmashankar K, Widlansky ME. 2010 Vascular endothelial function and hypertension: insights and directions. Curr. Hypertens. Rep. **12**, 448–455. (10.1007/s11906-010-0150-2) 20857237 PMC2982873

[54] Barker DJ, Osmond C. 1988 Low birth weight and hypertension. BMJ **297**, 134–135. (10.1136/bmj.297.6641.134-b) PMC1833827 3408942

[55] Wilde E, Aubdool AA, Thakore P, Baldissera L Jr, Alawi KM, Keeble J, Nandi M, Brain SD. 2017 Tail‐cuff technique and its influence on central blood pressure in the mouse. J. Am. Heart Assoc **6**, e005204. (10.1161/jaha.116.005204) 28655735 PMC5669161

[56] Walkowska A, Kuczeriszka M, Sadowski J, Olszyñski KH, Dobrowolski L, Červenka L, Hammock BD, Kompanowska-Jezierska E. 2015 High salt intake increases blood pressure in normal rats: putative role of 20-HETE and no evidence on changes in renal vascular reactivity. Kidney Blood Press. Res. **40**, 323–334. (10.1159/000368508) 26067851 PMC4583220

[57] Lawes CM, Hoorn SV, Rodgers A. 2008 Global burden of blood-pressure-related disease, 2001. Lancet **371**, 1513–1518. (10.1016/s0140-6736(08)60655-8) 18456100

[58] Forouzanfar MH *et al*. 2015 Global, regional, and national comparative risk assessment of 79 behavioural, environmental and occupational, and metabolic risks or clusters of risks in 188 countries, 1990–2013: a systematic analysis for the global burden of disease study 2013. Lancet **386**, 2287–2323. (10.1016/s0140-6736(15)00128-2) 26364544 PMC4685753

[59] Lackland DT, Weber MA. 2015 Global burden of cardiovascular disease and stroke: hypertension at the core. Can. J. Cardiol. **31**, 569–571. (10.1016/j.cjca.2015.01.009) 25795106

[60] Pilic L, Pedlar CR, Mavrommatis Y. 2016 Salt-sensitive hypertension: mechanisms and effects of dietary and other lifestyle factors. Nutr. Rev. **74**, 645–658. (10.1093/nutrit/nuw028) 27566757

[61] Nadruz W. 2015 Myocardial remodeling in hypertension. J. Hum. Hypertens. **29**, 1–6. (10.1038/jhh.2014.36) 24804791

[62] Baldo MP, Rodrigues SL, Mill JG. 2015 High salt intake as a multifaceted cardiovascular disease: new support from cellular and molecular evidence. Heart Fail. Rev. **20**, 461–474. (10.1007/s10741-015-9478-7) 25725616

[63] Dokainish H. 2015 Left ventricular diastolic function and dysfunction: central role of echocardiography. Glob. Cardiol. Sci. Pract. **2015**, 3. (10.5339/gcsp.2015.3) 25830147 PMC4374097

[64] Cho JH, Zhang R, Kilfoil PJ, Gallet R, de Couto G, Bresee C, Goldhaber JI, Marbán E, Cingolani E. 2017 Delayed repolarization underlies ventricular arrhythmias in rats with heart failure and preserved ejection fraction. Circulation **136**, 2037–2050. (10.1161/circulationaha.117.028202) 28974519 PMC5698112

[65] Yu HCM, Burrell LM, Black MJ, Wu LL, Dilley RJ, Cooper ME, Johnston CI. 1998 Salt induces myocardial and renal fibrosis in normotensive and hypertensive rats. Circulation **98**, 2621–2628. (10.1161/01.cir.98.23.2621) 9843472

[66] Galli G, Zi M, Baba H, Ashton N. 2025 Effects of a postnatal high-salt diet on cardiac function in offspring from hypoxic pregnancies. Figshare. (10.6084/m9.figshare.28143056) PMC12368548 40836814

[67] Baba H, Zi M, Ashton N, Galli GL. 2025 Supplementary material from: Effects of a postnatal high-salt diet on cardiac function in offspring from hypoxic pregnancies. Figshare. (10.6084/m9.figshare.c.7901685) PMC12368548 40836814

